# Lack of SARS Transmission among Public Hospital Workers, Vietnam

**DOI:** 10.3201/eid1002.030707

**Published:** 2004-02

**Authors:** Le Dang Ha, Sharon A. Bloom, Nguyen Quang Hien, Susan A. Maloney, Le Quynh Mai, Katrin C. Leitmeyer, Bach Huy Anh, Mary G. Reynolds, Joel M. Montgomery, James A. Comer, PeterW. Horby, Aileen J. Plant

**Affiliations:** * Institute for Clinical Research in Tropical Medicine, Hanoi, Vietnam; † Bach Mai Hospital, Hanoi, Vietnam; ‡ Centers for Disease Control and Prevention, Atlanta, Georgia, USA; § National Institute for Hygiene and Epidemiology, Hanoi, Vietnam; ¶ Communicable Disease Surveillance and Response, World Health Organization (WHO), Geneva, Switzerland; # Hanoi Medical University, Hanoi, Vietnam; ** Communicable Disease Surveillance and Response, WHO, Hanoi, Vietnam; †† Curtin University of Technology, Perth, Australia; 1 Primary coauthor.

**Keywords:** severe acute respiratory syndrome, Vietnam, health care personnel

## Abstract

The severe acute respiratory syndrome (SARS) outbreak in Vietnam was amplified by nosocomial spread within hospital A, but no transmission was reported in hospital B, the second of two designated SARS hospitals. Our study documents lack of SARS-associated coronavirus transmission to hospital B workers, despite variable infection control measures and the use of personal protective equipment.

Vietnam was one of the first countries affected by the global severe acute respiratory syndrome (SARS) outbreak and on April 28, 2003, was the first country to be removed from the World Health Organization (WHO) list of SARS-affected countries. Sixty-one patients with laboratory-confirmed SARS were hospitalized in two hospitals, six of whom died; including the index case-patient. All case-patients were epidemiologically-linked to the index case-patient, and most outbreak amplification occurred within one hospital. We investigated whether nosocomial transmission occurred among healthcare workers in the second hospital.

## The Study

The SARS outbreak in Vietnam began with the admission of a traveler from Hong Kong on February 26, 2003, to hospital A, a 56-bed, three-story, privately owned and expatriate-operated facility located in Hanoi. Within 2 weeks, extensive nosocomial transmission of SARS occurred in workers, patients, and visitors in hospital A. On March 12, hospital A was closed to new admissions except for sick hospital A workers. On that date, the 120-bed, six-story public hospital B began admitting patients with suspected and probable SARS. Hospital B treated 33 patients with laboratory-confirmed SARS between March 12 and May 2, 2003, the discharge date of the last patient (Figure). Of these, 23 were admitted directly to hospital B, and 10 were transferred from hospital A to hospital B on March 28. Many of hospital B’s 33 patients were exposed to SARS as patients or visitors in hospital A.

**Figure:**
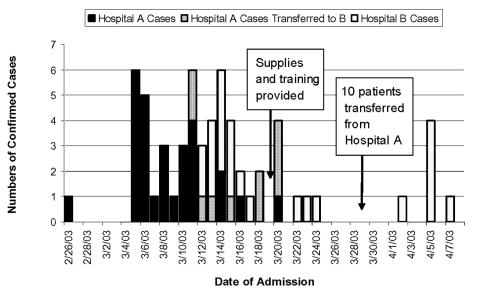
Laboratory-confirmed cases of severe acute respiratory syndrome (SARS) by date of admission, in hospital A and hospital B, Vietnam, February–April 2003. The ten case-patients who were transferred from hospital A to hospital B on March 29, 2003, are noted by cross-hatching.

No nosocomial SARS-associated coronavirus (SARS-CoV) transmission was reported in hospital B, and none of its 117 healthcare workers (defined as all staff working in the hospital building during the SARS outbreak) became ill with a SARS-compatible illness. This situation occurred despite obvious challenges to infection control. When hospital B began admitting patients, visitors were not tightly restricted, the main elevator was out of service, and families and workers often used the designated patient elevator. Researchers (K.C.L., H.Q.N.) and infection control advisors working daily on the hospital B wards reported variable infection control and patient isolation, particularly during the early weeks. On March 19, formal infection control training was organized and substantial technical support and supplies arrived from WHO, Médecins Sans Frontières–Belgium, and the Japan International Cooperation Agency. Systems were established to restrict visitors, and entry guards and Médecins Sans Frontières’ advisors were tasked with distributing and monitoring personal protective equipment, such as N95 masks, gloves, gowns, and hand sanitizer. Two of the authors of this article (K.C.L., H.Q.N.), who worked daily on the wards, observed that infection control practices improved considerably after these interventions.

To help researchers determine whether SARS-CoV transmission occurred among hospital B healthcare workers, staff were offered serologic testing from May 12 to 14 and were asked to complete a short questionnaire in Vietnamese. Participants provided written consent and answered questions about demographics, level of contact with SARS case-patients, and personal protective equipment use during the busiest week of patient admissions (March 12–19) and the remaining weeks of the outbreak. Serum specimens were analyzed at the National Institute for Hygiene and Epidemiology, Hanoi, and at the Centers for Disease Control and Prevention, Atlanta, by indirect enzyme-linked immunosorbent assay (ELISA) and indirect immunofluorescence (IFA) on Vero E-6 cells infected with SARS-CoV (*1*). Data were double-entered into Excel and analyzed with SAS Version 8.0 (SAS, Inc., Cary, NC).

Of 117 hospital B healthcare workers, 108 participated (92.3% response rate). According to the hospital director, all 9 nonparticipants remained well, and none had a history of SARS-like illness. Among participants, 62 (57.4%) respondents worked on the SARS wards (Table). Most (85.5%) were physicians and nurses. During the first week of SARS patient care in hospital B, 39 (62.9%) of SARS ward workers reported working in SARS-patient rooms for >6 hours on their single busiest day. Of the 62 workers, 58.1% and 64.5% reported being in SARS patient rooms during medication nebulizer treatment, and 65% reported being in patient rooms during noninvasive positive pressure ventilation.

**Table:** Occupations, SARS exposures, symptoms, and personal protective equipment use among workers on the SARS wards, hospital B, Vietnam, May 2003^a,b^

	SARS ward respondents N (%)	
Occupation:		
Physicians	23 (37.1)	
Nurses	30 (48.4)	
Nonclinical staff (housekeepers, clerks, elevator operators, laboratory technicians, and guards)	9 (14.5)	
Ever in room while SARS patient getting nebulized medications	36 (58.1)	
Ever in room while SARS patient receiving noninvasive positive pressure ventilation	40 (64.5)	
During the first week of SARS patient care (March 12–19):		
On busiest day, worked >6 hours in SARS patient’s room	39 (62.9)	
Wore a mask in patient’s room “always” or “usually”	61 (98.4)	
Wore only cloth mask, surgical mask, or both	43 (69.4)	
Wore N-95 mask and other type of mask	19 (30.6)	
Wore gloves in patient room “always” or “usually”	48 (77.4)	
After first week of SARS patient care:		
Wore face mask in patient’s room “always” or “usually”	56 (90.3)	
Wore N95 mask	62 (100)	
Wore gloves in patient’s room “always” or “usually”	47 (75.8)	
	
Symptoms and personal health behaviors:		
Fever	1 (1.6)	
Cough	10 (16.1)	
Sore throat	16 (22.6)	
Extreme fatigue	31 (50)	

^a^SARS, severe acute respiratory syndrome. ^b^N = 62

All 62 SARS ward workers reported wearing masks during the outbreak. All but one respondent wore a mask “always” or “usually” while in SARS patients’ rooms. However, during the first week of SARS patient care in hospital B, 43 ward workers (69.4%) reported wearing only a cloth or surgical mask, often in combination. All 62 SARS ward workers reported using an N-95 mask after March 19, although only 56 (90.3%) reported “always” or “usually” using a mask while in SARS patients’ rooms. Respondents reported using gloves 77.4% of the time before March 19 and 75.8% after March 19.

Reported symptoms and personal health behaviors of healthcare workers are also presented in the Table. One SARS ward respondent reported a fever, and less than 23% reported either a cough or sore throat. Extreme fatigue was reported by 50% of the SARS ward workers. Antibodies to SARS-CoV among our study participants were undetectable by both laboratories.

## Conclusions

This study has several limitations. First, our survey is subject to recall and reporting bias, because not only was it difficult for respondents to recall behaviors during specific periods within the previous 2 months, but respondents may have been concerned that results could be used to evaluate their performance. Estimates of SARS exposures and the frequency of personal protective equipment use among SARS ward workers are therefore probably inflated. Second, we collected serum specimens approximately 10 to 12 days after the last SARS patients were discharged; although these patients were discharged after their 5th to 6th week of illness, the minimal chance that a patient shed virus beyond the usual 2- to 3-week period (*2*) would theoretically mean that a few participants may have been tested before seroconversion. A third limitation is our lack of data on hand-washing or sanitizing practices, important means of preventing respiratory virus droplet spread.

The finding of no infection with SARS-CoV among hospital B workers in the presence of 33 confirmed SARS case-patients may support the hypothesis that, in the absence of a superspreading patient or event, most SARS patients will not transmit the virus (*3*–*6*). For example, in Singapore, 81% of the first 205 reported probable case-patients had no evidence of transmission of clinically identifiable SARS to other persons (*3*). Over 35 healthcare workers in our study reported being exposed to a SARS patient during events that can potentially generate aerosols (i.e., nebulizer treatment or noninvasive positive pressure ventilation), yet they did not acquire SARS. Although likely many factors contributed, we demonstrated a lack of SARS transmission both before and after the provision of formal infection control training and personal protective equipment. Contrasting the hospital B situation with that of neighboring hospital A may be helpful; in hospital A, extensive transmission clusters followed admission of the index case-patient.

The 23 directly admitted hospital B patients were less severely ill than the 38 hospital A patients. In Vietnam, the best available measure of relative disease severity is the death rate and the maximal level of respiratory assistance provided. Although no hospital B patients died or received invasive mechanical ventilation, four received biphasic intermittent positive airway pressure. Seven hospital A case-patients were intubated; an additional two received biphasic intermittent positive airway pressure. Five hospital A case-patients died in Vietnam, and the index case-patient died in Hong Kong (*7*).

Hospital A workers did not wear masks in the earliest days after the index case-patient was admitted, although shortly after the recognition of this nosocomial cluster, enhanced infection control measures were initiated. In contrast, by the time patients were going to hospital B for evaluation, both patients and healthcare workers were wearing masks (N.T. Van, pers. comm.)

Hospital A nursing staff likely also had longer and closer contact with SARS patients. In nursing style, hospital B resembled those of other public hospitals in Vietnam, where nursing is traditionally a shared function with family members. Families of SARS patients in hospital B were observed by authors (K.C.L., N.Q.H.) to be feeding, bathing, and toileting the patients. Hospital A nurses, however, were required by hospital guidelines to assume most patient care functions traditionally shared with the patient’s family (L.T. Hong, pers. comm.), thereby increasing their direct contact with SARS patients and their respiratory and other secretions. Furthermore, the more severely ill SARS patients of hospital A likely required more intensive nursing care, perhaps increasing the duration and dose of SARS-CoV exposure.

Environmental conditions at the two hospitals differed, but the impact of these differences on SARS transmission is unclear. Neither hospital had negative pressure rooms. Hospital A was a more modern facility; however, hospital B had designated SARS isolation wards and large spacious rooms with high ceilings and ceiling fans and large windows kept open for cross-ventilation. In contrast, hospital A’s rooms were smaller, and individual air-conditioning units were in use early during the outbreak. In addition, hospital A had diverse patients (maternity, postoperative, pediatric, etc.) housed on the same hospital floor when the SARS outbreak began.

The findings of lack of transmission among hospital B healthcare workers raises the question of whether family caregivers or visitors might have become infected with SARS-CoV, and about the relative infectiousness of hospital B patients in general. Although overt SARS transmission to visitors occurred in hospital A, no such transmission to visitors was observed in Hospital B. We lack adequate data to quantify the exposure of visitors to patients at either hospital, but the authors who were present (K.C.L., H.Q.N.) noted that after the first week, most Hospital B family members tended to always wear masks and to rarely use gloves. Studies assessing the serologic status of family and community contacts of case-patients are ongoing. Although community transmission did not seem to play a major role in the Vietnam SARS outbreak, at least two episodes are known in which SARS transmission occurred outside the hospital setting. One episode involved transmission from a visitor to hospital A to five contacts. This visitor was severely ill and was later hospitalized at hospital B on day 10 after symptom onset; he is known to have transmitted infection to one contact in the 4 hours immediately before his admission. If SARS viral shedding peaks on day 10 of illness and continues for 2–3 weeks (*3*), we can assume that some of the hospital B patients were still infectious during their hospitalization. Among the 23 directly admitted hospital B patients, the median days to admission was 7 (range 1–13) after illness onset.

In conclusion, we found no evidence of SARS-CoV transmission among hospital B workers, despite contact with laboratory-confirmed SARS case-patients and variable infection control practices and use of personal protective equipment. This finding may be explained by differences in infection control practices, use of personal protective equipment (including masks for patients as well as healthcare workers), nursing style, environmental features, and clinical factors such as severity of illness and the absence of a highly infectious SARS-CoV spreader. More study is needed to determine how each of these factors affects the risk of SARS transmission if we are to adequately prepare for future SARS epidemics.
